# Altered skeletal muscle fatty acid handling is associated with the degree of insulin resistance in overweight and obese humans

**DOI:** 10.1007/s00125-016-4104-3

**Published:** 2016-09-15

**Authors:** Birgitta W. van der Kolk, Gijs H. Goossens, Johan W. Jocken, Ellen E. Blaak

**Affiliations:** Department of Human Biology, NUTRIM School of Nutrition and Translational Research in Metabolism, Maastricht University Medical Center+, PO Box 616, 6200 MD Maastricht, the Netherlands

**Keywords:** Chylomicrons, Insulin resistance, Lipid metabolism, Skeletal muscle, VLDL

## Abstract

**Introduction/hypothesis:**

Disturbances in skeletal muscle fatty acid (FA) handling may contribute to the development and progression of whole-body insulin resistance (IR). In this study, we compared fasting and postprandial skeletal muscle FA handling in individuals with varying degrees of IR.

**Methods:**

Seventy-four overweight/obese participants (62 men) were divided into two groups based on the HOMA-IR median (3.35). Fasting and postprandial skeletal muscle FA handling were determined by combining the forearm muscle balance technique with stable isotopes. [^2^H_2_]palmitate was infused i.v. to label VLDL-triacylglycerol (VLDL-TAG) and NEFA in the circulation, whereas [U-^13^C]palmitate was incorporated in a high-saturated FA mixed-meal labelling chylomicron-TAG. Skeletal muscle biopsies were taken to assess intramuscular lipid content, fractional synthetic rate (FSR) and the transcriptional regulation of FA metabolism.

**Results:**

Postprandial forearm muscle VLDL-TAG extraction was elevated in the high-IR vs the mild-IR group (AUC_0-4h_: 0.57 ± 0.32 vs −0.43 ± 0.38 nmol [100 ml tissue]^−1^ min^−1^, respectively, *p* = 0.045). Although no differences in skeletal muscle TAG, diacylglycerol, NEFA content and FSR were present between groups, the high-IR group showed increased saturation of the intramuscular NEFA pool (*p* = 0.039). This was accompanied by lower muscle *GPAT1* (also known as *GPAM*) expression (*p* = 0.050).

**Conclusions/interpretation:**

Participants with high-IR demonstrated increased postprandial skeletal muscle VLDL-TAG extraction and higher saturation of the intramuscular NEFA pool vs individuals with mild-IR. These data support the involvement of disturbances in skeletal muscle FA handling in the progression of whole-body IR.

## Introduction

Systemic lipid overflow, which is driven by adipose tissue dysfunction and impaired skeletal muscle lipid handling, is associated with insulin resistance (IR) [1]. Increased circulating triacylglycerol (TAG) and NEFA concentrations are often found in IR because of impaired adipose tissue lipid handling [2–4]. This results in an increased lipid supply to other non-adipose tissues, such as liver and skeletal muscle. Due to an impaired capacity to oxidise fatty acids (FAs) [5, 6], these lipids may accumulate and interfere with insulin signalling in the liver and skeletal muscle [7, 8]. Over the last decade, it has become clear that the amount of lipids per se does not determine IR. Rather, a complex interplay between FA supply, FA type, muscle lipid turnover, subcellular localisation and composition of specific bioactive lipid metabolites seems to determine skeletal muscle IR [1, 8, 9].

The contribution of dietary fat (chylomicron-TAG) and endogenous fat (NEFA and VLDL-TAG) to skeletal muscle FA handling is not well understood. Elevated plasma NEFA concentrations may result from both expanded fat mass [2] and reduced peripheral clearance [5, 10]. Despite a reduced lipolysis per unit fat mass because of hyperinsulinaemia [11, 12], the total amount of NEFA released from adipose tissue in the postprandial state seems to be elevated in the obese insulin resistant state [13]. Furthermore, in situations with plasma insulin concentrations comparable to insulin levels achieved during a hyperinsulinaemic–euglycaemic clamp, the spillover from FAs derived from lipoprotein lipase (LPL)-mediated TAG hydrolysis in adipose tissue has been shown to be less suppressed in obese patients with type 2 diabetes than in non-obese healthy controls [14]. This increase in adipose tissue NEFA output might lead to increased hepatic VLDL-TAG production and elevated plasma TAG concentrations [15, 16]. Bickerton et al [17] demonstrated that dietary FAs were preferentially taken up in adipose tissue and skeletal muscle in the postprandial state in healthy lean humans, even though VLDL particles were abundantly present after the meal [11].

Up to now, most studies investigating combined VLDL- and chylomicron-TAG metabolism have been performed in healthy, lean humans [17, 18]. Bickerton et al [11] have shown elevated postprandial plasma VLDL- and chylomicron-TAG concentrations in overweight men with IR [11]. Moreover, we have recently demonstrated that an increased postprandial VLDL-TAG extraction was associated with IR in men with the metabolic syndrome [19]. However, studies involving the combined assessment of human skeletal muscle VLDL- and chylomicron-TAG metabolism, and intramuscular lipid species are limited. In addition, previous studies have used a relatively small sample size due to methodological difficulties as well as the high costs associated with these measurements. Therefore, extensive human in vivo data on skeletal muscle FA handling in IR are currently lacking. The aim of this study was to investigate fasting and postprandial skeletal muscle FA handling in a large study cohort of overweight or obese participants with a wide range of IR. A dual stable isotope tracer technique using labelled palmitate in combination with measurements of differences in arteriovenous concentrations across forearm muscle and forearm blood flow was used in this study, as previously validated [17]. This enabled us to differentiate between the metabolic fate of dietary and endogenous FA. In addition, skeletal muscle biopsies were taken to investigate skeletal muscle lipid metabolites, their fractional synthetic rates (FSRs) and the transcriptional regulation of FA metabolism.

## Methods

### Study participants

Seventy-four participants (62 men and 12 women) with the metabolic syndrome or impaired glucose metabolism were obtained from the Maastricht biobank. These participants (described elsewhere in more detail [19–21]) underwent a high-saturated FA (SFA) mixed-meal test. Participants were divided into two groups based on the median of HOMA-IR (3.35); participants below the median of HOMA-IR formed the ‘mild-IR’ group (*n* = 37) and participants above the median formed the ‘high-IR’ group (*n* = 37). The local Medical Ethical Committee of Maastricht University Medical Center^+^ approved the study protocols. All participants gave their written informed consent before participation.

### High-fat mixed-meal test

Participants were studied after an overnight fast and were asked to refrain from strenuous exercise and drinking alcohol for 24 h before the study day. In addition, they were asked to avoid food products naturally enriched with ^13^C for 7 days before the study day. Forearm muscle metabolism was studied using arteriovenous concentration differences combined with measurements of forearm blood flow. Three catheters were inserted before the start of the experiment. One catheter was placed retrogradely into a superficial dorsal vein of a hand heated in a hot-box (60°C) to obtain an arterialised blood sample. In the same arm another catheter was placed in an antecubital vein for the infusion of the [^2^H_2_]palmitate tracer. A third catheter was placed retrogradely in a deep antecubital vein of the contralateral forearm to sample venous blood draining the forearm muscle. After taking an arterialised and deep-venous background sample at 90 min before meal ingestion, a continuous i.v. infusion of the stable isotope tracer, [^2^H_2_]palmitate (97% enrichment; Cambridge Isotope Laboratories, Andover, MA, USA) complexed to albumin was started (0.035 μmol [kg body weight]^−1^ min^−1^). Baseline blood sampling was started after 1 h of tracer infusion to allow for isotopic equilibration to occur. Blood samples were taken simultaneously from the dorsal hand vein and the deep muscle vein at three time points during fasting. Samples were also taken at six time points postprandially after consumption of a high-SFA mixed-meal (at ‘0 min’) containing 200 mg [U-^13^C]palmitate (98% enrichment; Cambridge Isotope Laboratories). The liquid meal provided 2.6 MJ energy, consisting of 61 energy % (E%) fat (35.5 E%, SFA; 18.8 E%, monounsaturated FA [MUFA]; 1.7 E% polyunsaturated FA [PUFA]), 33 E% carbohydrates and 6.3 E% protein. Analysis of forearm blood flow before blood sampling and details of other biochemical analyses have been described previously [19].

### Skeletal muscle biopsies

Skeletal muscle biopsies were obtained from the vastus lateralis muscle after local anaesthesia of the skin and fascia using the Bergström method with suction [22]. Muscle biopsies were taken during fasting and at the end of the postprandial period (240 min). Muscle biopsies were lyophilised and dissected free of extramyocellular lipid, blood and connective tissue under a microscope. Details of lipid extraction and quantification have been described previously [19]. Skeletal muscle expression of genes related to transcription factors, oxidative metabolism, lipid synthesis and lipolysis were analysed. Gene expression was normalised relative to the geometric mean of the internal reference genes (β-actin, β-2-microglobulin and/or ribosomal protein L13a). Details of accession numbers, RNA primer sequences and RT-PCR analysis of these genes have been described previously [19, 20]. Since limited muscle biopsy samples were available, not all genes have been measured in all individuals.

### Calculations

Net fluxes of metabolites (labelled and unlabelled) across the forearm were calculated by multiplying the arteriovenous concentration difference by forearm plasma flow. Plasma flow was measured using plethysmography and calculated by multiplying forearm blood flow with ([1-haematocrit (%vol.)]/100). A positive flux indicates net uptake across forearm muscle, whereas a negative flux indicates net release. Labelled NEFA and TAG concentrations were calculated as the product of tracer:tracee ratio (TTR) of [^2^H_2_]palmitate and [U-^13^C]palmitate and the concentration of palmitate in NEFA and TAG, as reported previously [19].

The degree of saturation of skeletal muscle TAG, diacylglycerol (DAG), phospholipid (PL) and NEFA (%) was calculated by dividing the sum of unsaturated FAs by the total amount of FAs in a fraction multiplied by 100. The FSR of skeletal muscle NEFA, TAG, DAG and PL was calculated using skeletal muscle NEFA as the precursor pool for lipid synthesis. The increase in TTR of [U-^13^C] from fasting to 4 h postprandial measures was divided by the enrichment of skeletal muscle NEFA and expressed as per cent per hour (%/h). Postprandial areas under the curve (AUC_0-4h_) of metabolites were calculated using the trapezium rule and in this study data are presented as AUC_0-4h_/min.

### Statistics

In this study all data are expressed as mean ± SEM. Participant characteristics and differences in skeletal muscle lipid handling during fasting and postprandial conditions of the mild-IR and high-IR group were compared using independent samples *t* tests. A linear regression was performed with HOMA-IR and BMI and sex as co-variates. Variables were log_e_-transformed if the assumption of normality was not met. The data were analysed using SPSS for Mac version 22.0 (SPSS, Chicago, IL, USA) and statistical significance was set at *p* < 0.05.

## Results

### Study population

Participant characteristics are summarised in Table 1. Age, waist:hip ratio and blood pressure were comparable between groups, while BMI was significantly higher in the high-IR group (*p* = 0.002). By design, mean HOMA-IR was different between the two groups (mild-IR vs high-IR: 2.5 ± 0.1 vs 4.7 ± 0.3, respectively, *p* < 0.001).

**Table 1:** Characteristics of participants

Characteristic	Mild-IR (*n* = 37)	High-IR (*n* = 37)	Total group (*n* = 74)	Range (*n* = 74)	*p* value^a^
Male (*n*)/Female (*n*)	33/4	29/8	62/12		
Age (years)	58.0 ± 1.4	59.0 ± 1.1	58.5 ± 0.9	36–70	0.582
Body weight (kg)	89.8 ± 1.9	95.1 ± 2.1	92.5 ± 1.4	64.0–115.0	0.064
BMI (kg/m^2^)	29.2 ± 0.5	31.7 ± 0.6	30.5 ± 0.4	22.7–39.5	0.002
Waist:hip ratio	1.02 ± 0.01	1.03 ± 0.01	1.02 ± 0.01	0.89–1.17	0.494
Systolic blood pressure (mmHg)	135 ± 2	136 ± 2	135 ± 2	105–174	0.636
Diastolic blood pressure (mmHg)	84 ± 2	85 ± 1	85 ± 1	68–110	0.944
Fasting plasma glucose (mmol/l)	5.4 ± 0.1	5.7 ± 0.1	5.5 ± 0.1	4.6–6.7	0.002
Fasting plasma insulin (pmol/l)	72.3 ± 2.4	130.2 ± 6.6	101.2 ± 4.9	50–263.3	<0.001
Fasting plasma NEFA (μmol/l)	561 ± 22	598 ± 26	580 ± 17	296–1002	0.287
Fasting plasma TAG (μmol/l)	1320 ± 103	1246 ± 85	1283 ± 66	415–3232	0.582
HOMA-IR	2.5 ± 0.1	4.7 ± 0.3	3.6 ± 0.2	1.68–11.3	<0.001

Values are presented as mean ± SEM

^a^ *p* value for difference between mild-IR and high-IR group, Student’s *t* test for unpaired samples

### Arterialised metabolites, forearm muscle metabolism and forearm blood flow

**Fig. 1:**
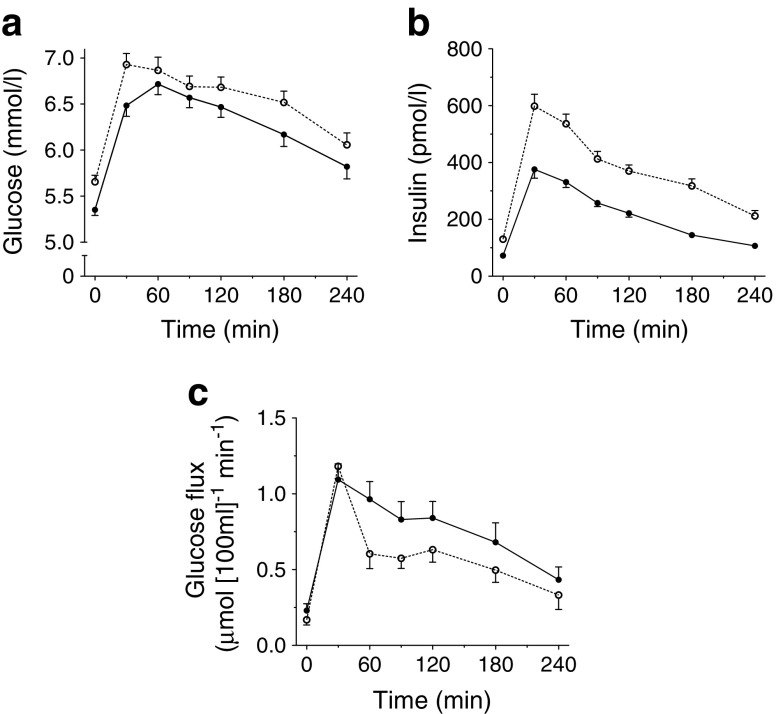
Arterialised plasma glucose (**a**) and insulin (**b**) concentrations and glucose flux (**c**) during fasting (0 min) and after consumption of a high-SFA meal. Black circles, mild-IR group; white circles, high-IR group. Student’s *t* test for unpaired samples showed significant effect of group on fasting and postprandial plasma glucose (*p* = 0.002 and AUC_0-4h_: *p* = 0.035, respectively), fasting and postprandial plasma insulin (*p* < 0.001 and AUC_0-4h_: *p* < 0.001, respectively) and postprandial glucose flux (AUC_0-4h_: *p* = 0.034). Values are presented as mean ± SEM

Fasting arterialised plasma glucose (Fig. 1a) and insulin (Fig. 1b) concentrations were significantly higher in the high-IR group than in the mild-IR group (*p* = 0.002 and *p* < 0.001, respectively) and remained higher throughout the postprandial period (*p* = 0.035 and *p* < 0.001, respectively). Net glucose uptake across forearm muscle was similar in the high-IR and mild-IR groups under fasting conditions, but was significantly lower in the high-IR group than in the mild-IR group after meal ingestion (AUC_0-4h_ 0.59 ± 0.04 vs 0.76 ± 0.07 μmol [100 ml tissue]^−1^ min^−1^, respectively, *p* = 0.034), indicating a lower postprandial insulin sensitivity in the high-IR group (see Table 2 and Fig. 1c). Arterialised fasting plasma glycerol concentrations (*p* = 0.006) and lactate concentrations were significantly higher (*p* = 0.006) in the high-IR group than in the mild-IR group (Table 2). Forearm blood flow and glycerol release were similar between the two IR groups, both during fasting and postprandial conditions. Moreover, fasting (*p* = 0.002) and postprandial (*p* = 0.048) lactate release was positively associated with IR (Table 2).

**Table 2:** Fasting and postprandial lipid metabolism

Variable	Mild-IR	High-IR	*p* value^a^	Total group SE β	*p* value^b^
Forearm blood flow (ml [100 ml tissue]^−1^ min^−1^)					
Fasting	2.5 ± 0.2	2.5 ± 0.2	0.896	0.054	0.682
Postprandial	2.6 ± 0.2	2.8 ± 0.2	0.476	0.118	0.369
Glycerol (μmol/l)					
Fasting	84.6 ± 3.9	118.5 ± 11.1	0.006	0.200	0.090
Postprandial	64.9 ± 3.6	92.9 ± 10.6	0.016	0.278	0.025
Lactate (μmol/l)					
Fasting	0.62 ± 0.04	0.79 ± 0.05	0.006	0.438	<0.001
Postprandial	0.98 ± 0.05	1.10 ± 0.05	0.081	0.224	0.087
Net flux across forearm muscle					
Glucose (μmol [100 ml tissue]^−1^ min^−1^)					
Fasting	0.23 ± 0.05	0.17 ± 0.03	0.274	−0.083	0.524
Postprandial	0.76 ± 0.07	0.59 ± 0.04	0.034	−0.147	0.226
Glycerol (nmol [100 ml tissue]^−1^ min^−1^)					
Fasting	−28.3 ± 5.5	−27.0 ± 7.7	0.883	0.224	0.092
Postprandial	−20.4 ± 4.5	−25.2 ± 6.5	0.541	0.081	0.543
Lactate (nmol [100 ml tissue]^−1^ min^−1^)					
Fasting	−0.18 ± 0.02	−0.12 ± 0.03	0.123	0.384	0.002
Postprandial	0.01 ± 0.02	0.04 ± 0.03	0.472	0.249	0.048
NEFA (nmol [100 ml tissue]^−1^ min^−1^)					
Fasting	−6.9 ± 20.4	26.6 ± 24.6	0.300	0.165	0.210
Postprandial	−0.8 ± 9.5	8.7 ± 13.4	0.565	0.112	0.405
[^2^H_2_]palmitate NEFA (nmol [100 ml tissue]^−1^ min^−1^)					
Fasting	1.69 ± 0.12	1.58 ± 0.14	0.539	0.017	0.902
Postprandial	1.50 ± 0.09	1.53 ± 0.14	0.846	0.171	0.208
TAG (nmol [100 ml tissue]^−1^ min^−1^)					
Fasting	10.2 ± 13.2	45.5 ± 12.1	0.052	0.094	0.466
Postprandial	53.9 ± 17.2	51.9 ± 28.4	0.954	−0.105	0.422
[^2^H_2_]palmitate TAG (nmol [100 ml tissue]^−1^ min^−1^)					
Fasting	−0.24 ± 0.16	0.56 ± 0.41	0.069	0.241	0.076
Postprandial	−0.43 ± 0.38	0.57 ± 0.32	0.045	0.294	0.037
[U-^13^C]palmitate TAG (nmol [100 ml tissue]^−1^ min^−1^)					
Fasting^c^					
Postprandial	0.86 ± 0.16	0.66 ± 0.16	0.353	−0.046	0.736

Values are presented as mean ± SEM

Postprandial values are calculated from AUC_0-4h_

A positive flux indicates net uptake across forearm muscle, whereas a negative flux indicates net release

^a^ *p* value for difference between mild-IR and high-IR group, Student’s *t* test for unpaired samples

^b^ *p* value for multiple linear regression

^c^[U-^13^C]palmitate was given with the high-SFA meal, hence fasting data are not available.

[^2^H_2_]palmitate TAG fasting, *n* = 34; [^2^H_2_]palmitate NEFA postprandial and [U-^13^C]palmitate TAG postprandial, *n* = 33; [^2^H_2_]palmitate TAG postprandial (mild-IR), *n* = 29; [^2^H_2_]palmitate TAG postprandial (high-IR), *n* = 32

SE β, standardised β coefficient for HOMA-IR, adjusted for sex and BMI

### Whole-body and forearm muscle NEFA metabolism

**Fig. 2:**
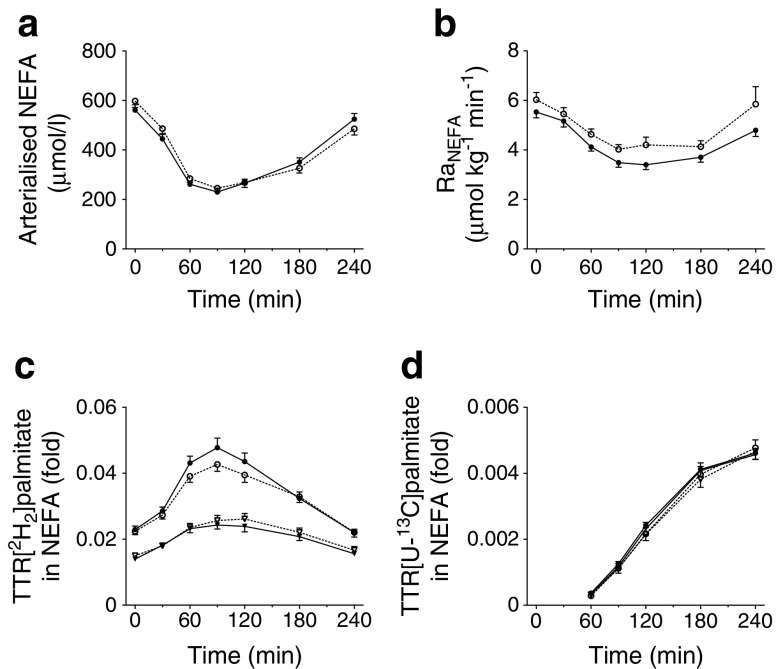
Postprandial whole-body NEFA metabolism. Arterialised plasma NEFA concentrations (**a**), Ra_NEFA_ (**b**), and the TTR of [^2^H_2_]palmitate (**c**) and [U-^13^C]palmitate (**d**) in the plasma NEFA fraction during fasting (0 min) and after consumption of a high-SFA meal. Black symbols, mild-IR group; white symbols, high-IR group; in (**c**) and (**d**) circles, arterialised plasma concentrations and triangles, forearm venous plasma concentrations. Values are presented as mean ± SEM

Fasting arterialised NEFA concentrations were similar in the high-IR and mild-IR groups (Fig. 2a). After the high-SFA meal ingestion, arterialised NEFA concentrations decreased to the same extent in both groups and returned to near-baseline values at the end of the postprandial period, with no significant differences observed between the groups. [^2^H_2_]palmitate was infused i.v. and was mixed with the plasma NEFA pool. The TTR reached steady state during fasting measurements (Fig. 2c, d). Consistent with these findings, the rate of appearance of NEFA (Ra_NEFA_) decreased after the meal (Fig. 2b), which is an indication of suppression of whole-body lipolysis. A reduction in postprandial suppression of the Ra_NEFA_ was observed in the high-IR group compared with the mild-IR group, although the difference did not reach statistical significance (*p* = 0.079, Fig. 2b). There were no differences in arterialised concentrations of [^2^H_2_]palmitate and [U-^13^C]palmitate in NEFA between groups (data not shown).

The TTR of [^2^H_2_]palmitate in NEFA was higher in arterialised vs deep-venous plasma at all time points in both groups. This reflects dilution of the [^2^H_2_] tracer in the plasma NEFA pool across forearm muscle. The TTR of [U-^13^C]palmitate in NEFA (resulting from spillover of FA derived from chylomicron-TAG hydrolysis) was not different in arterialised vs deep-venous plasma at all time points in both groups (Fig. 2c, d).

Fasting and postprandial net extraction of plasma NEFA across forearm muscle did not differ between groups. Furthermore, there was consistent uptake of [^2^H_2_]palmitate across forearm muscle during the study period and this was similar in both groups (Table 2).

### Whole-body and forearm muscle TAG metabolism

**Fig. 3:**
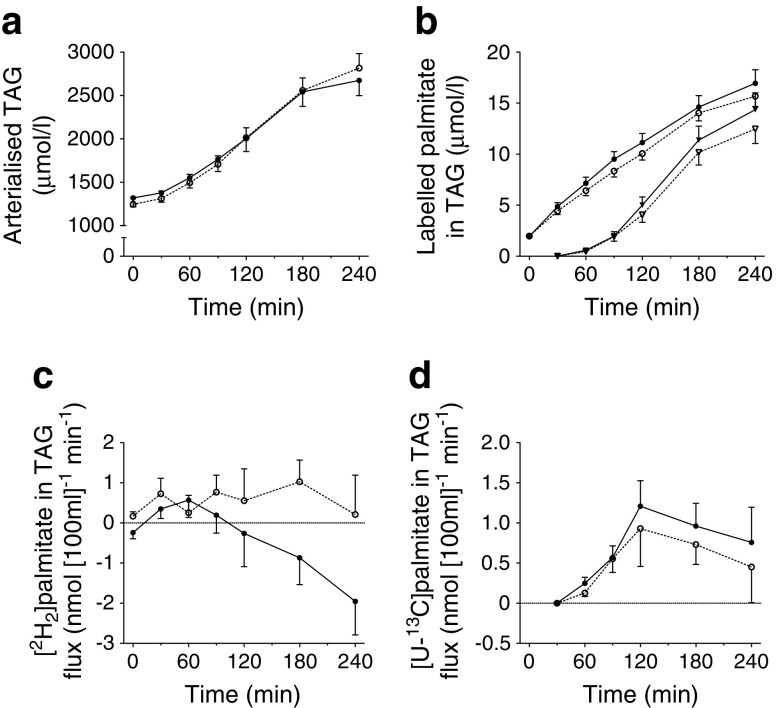
Postprandial whole-body and forearm muscle TAG metabolism. Arterialised plasma TAG concentrations (**a**), [^2^H_2_]- and [U-^13^C]palmitate concentrations in the plasma TAG fraction (**b**), and the net flux of [^2^H_2_]palmitate TAG (**c**) and [U-^13^C]palmitate TAG (**d**) across forearm muscle during fasting (0 min) and after consumption of a high-SFA meal. A positive flux indicates net uptake across forearm muscle, whereas a negative flux indicates net release. Black symbols, mild-IR group; white symbols, high-IR group; in (**b**) circles, [^2^H_2_]palmitate TAG and triangles, [U-^13^C]palmitate TAG. Student’s *t* test for unpaired samples showed a significant effect of group on postprandial net flux of [^2^H_2_]palmitate TAG (AUC_0-4h_; *p* = 0.045). Values are presented as mean ± SEM

Comparable arterialised TAG concentrations between the high-IR and mild-IR groups were observed during fasting and postprandial conditions (Fig. 3a). The [^2^H_2_]palmitate tracer was measurable in plasma TAG from the first baseline sample onwards, reflecting incorporation of the i.v. infused tracer into VLDL-TAG (Fig. 3b). The [U-^13^C]palmitate tracer, which was given with the meal, appeared in plasma TAG from 60 min after meal ingestion, representing chylomicron-TAG in the circulation (Fig. 3b). During the postprandial period both labelled TAG fractions increased without significant differences between groups.

Fasting net extraction of [^2^H_2_]palmitate TAG across forearm muscle was higher in the high-IR group than in the mild-IR group, although the difference did not reach statistical significance (*p* = 0.069). Postprandial net extraction of [^2^H_2_]palmitate TAG was significantly elevated in the high-IR group vs the mild-IR group (*p* = 0.045) (Fig. 3c). In line with this, we found a significant linear association between postprandial net extraction of [^2^H_2_]palmitate TAG and HOMA-IR, even after adjustment for BMI and sex (standardised β = 0.294; *p* = 0.037; Table 2). The net extraction of [U-^13^C]palmitate TAG across forearm muscle could be detected from 60 min onwards and did not differ significantly between groups (Table 2, Fig. 3d).

### Intramuscular lipid metabolism

**Fig. 4:**
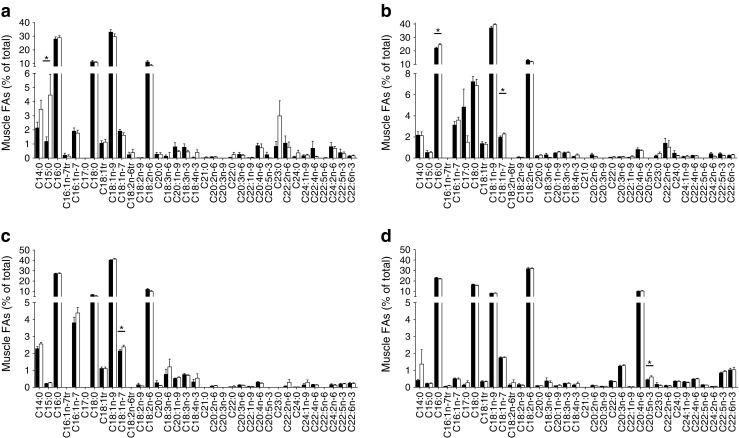
Intramuscular NEFA (**a**), DAG (**b**), TAG (**c**) and PL (**d**) composition under fasting conditions. Black bars, mild-IR group; white bars, high-IR group. *p < 0.05, mild-IR vs high-IR group, Student’s *t* test for unpaired samples. Values are presented as mean ± SEM

Skeletal muscle TAG, DAG and NEFA content were comparable between high-IR and mild-IR groups (Table 3). The PL content was lower in the high-IR group than in the mild-IR group, although the difference did not reach statistical significance (60.5 ± 3.2 vs 70.2 ± 3.6 μmol/[g dry weight], respectively, *p* = 0.055). However, there was no significant linear association between PL and measures of IR (Table 3). Intramuscular lipid composition was different between groups, with a significantly higher degree of saturation in the muscle NEFA pool in participants with high-IR compared with those with mild-IR (51.8 ± 2.8% vs 43.9 ± 2.5%, respectively, *p* = 0.039), but there was no significant linear association with IR (Table 3). This difference was mainly explained by higher percentages of myristic acid (C14:0; 3.5 ± 0.7% vs 2.1 ± 0.4%, *p* = 0.078), pentadecyclic acid (C15:0; 4.5 ± 1.5% vs 1.2 ± 0.3%, *p* = 0.038) and tricosylic acid (C23:0; 3.0 ± 1.1% vs 0.8 ± 0.4%, *p* = 0.064) in the high-IR group vs mild-IR group (Fig. 4a). Furthermore, the percentage of PUFA in the NEFA pool was lower in the high-IR group than in the mild-IR group (13.0 ± 1.1% vs 17.0 ± 1.4%, *p* = 0.034; Table 3). In addition, lipid composition in the intramuscular DAG pool differed between high-IR and mild-IR groups (Table 3): in the high-IR group, the percentage PUFA was significantly lower (*p* = 0.022). Linear regression showed a non-significant negative association between the percentage of PUFA in the DAG pool and HOMA-IR. Furthermore, a higher percentage of MUFA was observed in the DAG pool of the high-IR group compared with the mild-IR group (*p* = 0.052; Table 3). Although the total saturation of the lipid content in the DAG pool did not differ between groups (Table 3), the percentage of palmitate (C16:0) in the DAG pool of the high-IR group was significantly higher compared with that in the mild-IR group (22.1 ± 0.7% vs 24.6 ± 0.8%, respectively, *p* = 0.024; Fig. 4b).

**Table 3:** Skeletal muscle lipid content and composition during fasting and the FSR of the muscle lipid pools after a high-SFA meal

Lipid	Mild-IR (*n* = 35)	High-IR (*n* = 28)	*p* value^a^	Total group SE β	*p* value^b^
NEFA					
Total (μmol/[g dry weight])	5.9 ± 0.8	4.9 ± 0.8	0.360	−0.058	0.642
% SFA	43.9 ± 2.5	51.8 ± 2.8	0.039	0.219	0.112
% MUFA	39.1 ± 1.9	35.2 ± 2.3	0.183	−0.183	0.162
% PUFA	17.0 ± 1.4	13.0 ± 1.1	0.034	−0.162	0.266
FSR (%/h)	0.38 ± 0.05	0.36 ± 0.07	0.747	−0.069	0.619
DAG					
Total (μmol/[g dry weight])	10.8 ± 2.8	6.2 ± 0.7	0.151	−0.195	0.160
% SFA	37.8 ± 1.9	36.6 ± 0.8	0.594	−0.093	0.535
% MUFA	44.2 ± 1.6	47.6 ± 0.6	0.052	0.255	0.086
% PUFA	18.0 ± 0.8	15.7 ± 0.5	0.022	−0.271	0.059
FSR (%/h)	0.33 ± 0.05	0.29 ± 0.05	0.538	−0.058	0.692
TAG					
Total (μmol/[g dry weight])	182.6 ± 24.3	232.1 ± 42.5	0.301	0.126	0.365
% SFA	36.7 ± 0.9	35.8 ± 0.9	0.477	−0.157	0.273
% MUFA	47.9 ± 1.0	50.1 ± 0.7	0.076	0.046	0.749
% PUFA	15.3 ± 0.8	14.1 ± 1.0	0.325	0.112	0.444
FSR (%/h)	0.28 ± 0.05	0.21 ± 0.04	0.325	−0.186	0.209
PL					
Total (μmol/[g dry weight])	70.2 ± 3.6	60.5 ± 3.2	0.055	−0.155	0.260
% SFA	41.3 ± 0.8	40.4 ± 0.6	0.364	−0.046	0.754
% MUFA	11.3 ± 0.3	11.4 ± 0.5	0.903	0.017	0.909
% PUFA	47.4 ± 1.0	48.2 ± 0.7	0.496	0.030	0.838
FSR (%/h)	0.10 ± 0.01	0.09 ± 0.01	0.486	−0.006	0.966

Values are presented as mean ± SEM

^a^ *p* value for difference between mild-IR and high-IR group, Student’s *t* test for unpaired samples

^b^ *p* value for multiple linear regression

SE β, standardised β coefficient for HOMA-IR, adjusted for sex and BMI

The FSR of skeletal muscle TAG, DAG and PL was comparable between groups (Table 3), indicating that a similar proportion of palmitate from the intramuscular NEFA pool was directed towards storage after the high-SFA meal.

Fasting skeletal muscle mRNA expression of genes encoding proteins involved in oxidative metabolism, lipid synthesis and lipolysis is shown in Table 4. Skeletal muscle gene expression of *GPAT1* (also known as *GPAM*), a gene involved in lipid synthesis, was significantly lower (*p* = 0.050) in the high-IR group than in the mild-IR group, even when adjusted for sex and BMI using linear regression analysis (standardised β = −0.666; *p* = 0.002). Furthermore, the expression level of *NDUFB5*, encoding a subunit of complex I in the electron transport chain, tended to be lower in the high-IR vs the mild-IR group (*p* = 0.062), whilst the expression of the intracellular lipase gene *ATGL* (also known as *PNPLA2*) tended to be higher in the high-IR group (*p* = 0.052).

**Table 4:** Fasting skeletal muscle gene expression

Gene	Mild-IR	High-IR	*p* value^a^	Total group SE β	*p* value^b^
Oxidative metabolism	*n* = 32	*n* = 30			
*mCPT1B*	1.70 ± 0.14	1.74 ± 0.13	0.821	0.006	0.986
*PGC1α* (*PPARGC1A*)	0.51 ± 0.05	0.46 ± 0.04	0.428	0.027	0.847
*ACC2*	1.52 ± 0.14	1.59 ± 0.16	0.741	0.020	0.891
*SDHB*	2.40 ± 0.21	1.97 ± 0.22	0.153	−0.058	0.683
*NDUFB5*	2.67 ± 0.17	2.14 ± 0.23	0.062	−0.243	0.076
Transcription factors	*n* = 28	*n* = 23			
*PPARα* (*PPARA*)	1.63 ± 0.16	1.56 ± 0.11	0.743	0.034	0.808
*PPARδ* (*PPARD*)	0.36 ± 0.04	0.48 ± 0.07	0.114	0.175	0.259
*SREBP1c* (*SREBF1*)	0.92 ± 0.10	1.12 ± 0.14	0.238	0.028	0.840
*SREBP2* (*SREBF2*)	1.43 ± 0.15	1.86 ± 0.23	0.127	0.266	0.216
*ChREBP* (*CREBBP*)	1.02 ± 0.19	0.99 ± 0.15	0.906	−0.040	0.859
TAG synthesis	*n* = 10	*n* = 15			
*GPAT1* (*GPAM*)	2.55 ± 0.23	1.81 ± 0.25	0.050	−0.666	0.002
*DGAT1*	1.89 ± 0.19	1.77 ± 0.20	0.667	0.079	0.683
*DGAT2*	0.31 ± 0.05	0.25 ± 0.06	0.509	−0.103	0.671
TAG lipolysis	*n* = 15	*n* = 8			
*LPL*	0.50 ± 0.08	0.62 ± 0.11	0.342	0.018	0.927
*ATGL* (*PNPLA2*)	1.11 ± 0.17	2.52 ± 0.60	0.052	0.522	0.007
*HSL* (*LIPE*)	0.44 ± 0.19	0.74 ± 0.21	0.329	0.130	0.515

Values are presented as mean ± SEM

The italic entries are all gene symbols

^a^ *p* value for difference between mild-IR and high-IR group, Student’s *t* test for unpaired samples

^b^ *p* value for multiple linear regression

*m*CPT1b, muscle *CPT1B*; SE β, standardised β coefficient for HOMA-IR, adjusted for sex and BMI

## Discussion

The present study demonstrated that postprandial forearm muscle VLDL-TAG extraction was elevated in individuals with high-IR compared with mild-IR. This elevation in VLDL-TAG extraction was accompanied by increased saturation of the intramuscular NEFA pool. Both effects were independent of BMI. These data support the notion of an important role for disturbances in skeletal muscle FA handling in the progression of whole-body IR.

Disturbances in skeletal muscle FA handling have been implicated in the aetiology of IR and type 2 diabetes [1, 23]. Our group has recently demonstrated that higher postprandial plasma TAG concentrations and increased net TAG extraction across forearm muscle were accompanied by decreased postprandial insulin sensitivity in participants with impaired glucose metabolism compared with normal glucose tolerance [24]. Furthermore, we showed a higher postprandial VLDL-TAG extraction by skeletal muscle in men with IR compared with controls with the metabolic syndrome, matched for age and BMI, despite a similar TAG supply [19]. More recently, we demonstrated that increased muscle VLDL-TAG extraction and reduced lipid turnover of SFA, rather than DAG content, accompany the more pronounced IR observed in humans with impaired glucose tolerance (IGT) compared with impaired fasting glucose (IFG) [20].

The present data extend our previous observations regarding FA handling by showing that postprandial forearm muscle VLDL-TAG extraction was elevated in participants with high-IR when compared with those with mild-IR. We included participants who encompassed the entire spectrum of insulin sensitivity, from insulin-sensitive to very-insulin-resistant states (HOMA-IR, 1.7–11.3) and with a wide range of adiposity (BMI, 22.7–39.5 kg/m^2^). This allowed us to differentiate between the effect of obesity and IR per se, and our present findings confirm those of previous studies that have suggested the existence of a relationship between impaired skeletal muscle FA handling and IR [1, 8, 25]. Moreover, our data imply that IR is primarily responsible for increased postprandial forearm muscle VLDL-TAG extraction, since we observed a significant linear association between VLDL-TAG extraction and IR. A potential mechanism for increased skeletal muscle VLDL-TAG extraction in participants with high-IR may involve differential apolipoprotein composition of the VLDL particles [16]. For example, higher plasma apoCII:apoCIII ratios have been shown in diabetic individuals, compared with a control group [26]. This variation in lipid composition might lead to higher a susceptibility for lipid degradation by in vivo skeletal muscle LPL [27]. Of note, we did not perform measurements in the late postprandial phase. It has been shown that dietary FAs appear in VLDL-TAG from 2–3 h after meal ingestion, making it difficult to separate chylomicron- and VLDL-TAG in the late postprandial phase using the current dual isotope approach [17, 28, 29]. Therefore, we cannot exclude the possibility that the increased TAG extraction observed in high-IR participants may also extend to chylomicron-TAG. In this context, an impaired inhibitory effect of insulin on skeletal muscle LPL action in the high-IR group [30], or impaired FA uptake via membrane-associated carrier proteins like CD36 [31] might possibly explain the observed differences.

In addition to an increased skeletal muscle lipid uptake, we observed differences in the intramyocellular FA partitioning between groups. Reduced re-esterification of NEFA into TAG may expose muscle to excess NEFA concentrations and bioactive lipid metabolites that may interfere with insulin signalling [23]. In line with this, a reduced incorporation of NEFA into TAG in primary myotubes from obese individuals with type 2 diabetes has previously been shown [32], indicating that the ability to incorporate FAs into TAG is an intrinsic feature of human muscle cells that is reduced in individuals with type 2 diabetes. Moreover, in vitro work has shown that muscle cells incubated with palmitate incorporated more FA towards the DAG pool, while the unsaturated FAs were diverted towards storage in the TAG pool [33]. In the present study, an increased percentage of palmitate in the DAG pool in the participants with high-IR was observed. Together with the reduced expression of *GPAT1*, which is involved in the first step in TAG synthesis in muscle, this might indicate a retention of SFA in the NEFA pool and may explain the lower percentage of PUFA in the DAG pool in the individuals with high-IR vs mild-IR. More recently, we reported a reduced FSR of palmitate into intramuscular TAG and DAG in individuals with combined IGT and IFG compared with participants with isolated IFG [20]. This has also been shown in obese humans with impaired glucose metabolism [34]. However, in the present study we did not observe significant differences between the mild-IR and high-IR groups in the FSR of palmitate into intramuscular TAG and DAG. Nevertheless, these findings suggest that the postprandial incorporation of FAs in TAG or DAG is not affected by the degree of IR per se.

Strikingly, in this study the increased saturation in the NEFA pool was mainly confined to specific SFAs, namely myristic acid (C14:0), pentadecyclic acid (C15:0) and tricosylic acid (C23:0). Recently, plasma odd-chain SFAs were shown to be inversely associated with type 2 diabetes and coronary heart disease incidence in large epidemiological studies [35]. Interestingly, dairy products are known to be the most important source for C15:0 [36]. This would certainly suggest a contribution of dietary fat to the increased saturation of the NEFA pool, since it has been shown that FA composition of skeletal muscle reflects dietary FA composition [37]. However, data related to odd-chain FA metabolism in skeletal muscle are currently lacking and more research is needed to unravel the role of odd-chain FAs in intramyocellular lipid turnover and skeletal muscle IR. Moreover, it is important to note that the increased content of SFA in the high-IR group might be linked to an increased ceramide content and higher saturation in the long-chain fatty acyl-CoA. It has been shown that increased levels of the SFA palmitate drive ceramide synthesis [38]. However, we did not measure skeletal muscle ceramide levels or expression of related genes in the present study. Finally, previous studies have shown that lower percentages of PUFA in the plasma membrane are associated with IR [39, 40]. However, in this study the percentages of total PUFAs in the muscle PL pool were comparable between groups. It is important to note that we have not specifically measured muscle membrane PL content but rather total muscle PL content. Since most PLs are located mainly in the plasma membrane, the reduced total PL content might reflect a lower absolute amount of PUFA in the muscle membrane fraction and might therefore contribute to the worsening of IR in individuals with high-IR.

A limitation of the present study is that we did not perform a hyperinsulinaemic–euglycaemic clamp test to assess insulin sensitivity. Rather, we divided groups based on a surrogate marker of whole-body insulin sensitivity, namely HOMA-IR. Nevertheless, previous studies have shown strong correlations between HOMA-IR and peripheral insulin sensitivity, as measured by the gold-standard hyperinsulinaemic–euglycaemic clamp [41]. Importantly, in this study individuals in the high-IR group had a significantly lower postprandial net glucose uptake across forearm muscle as compared with the individuals with mild-IR, despite having significantly higher postprandial plasma insulin concentrations. Since skeletal muscle accounts for approximately 80% of insulin-mediated glucose uptake in humans [42], these data clearly indicate a more pronounced skeletal muscle IR in the individuals with high-IR as compared with those with mild-IR.

In conclusion, increased skeletal muscle VLDL-TAG extraction in the postprandial state and higher saturation of the intramuscular NEFA pool are associated with more pronounced IR. These data support an important role for disturbances in skeletal muscle FA handling in the progression of whole-body IR.

## Abbreviations

DAG: Diacylglycerol
FA: Fatty acid
FSR: Fractional synthetic rate
IFG: Impaired fasting glucose
IGT: Impaired glucose tolerance
IR: Insulin resistance
LPL: Lipoprotein lipase
MUFA: Monounsaturated fatty acid
PL: Phospholipid
PUFA: Polyunsaturated fatty acid
Ra_NEFA_: Rate of appearance of NEFA
SFA: Saturated fatty acid
TAG: Triacylglycerol
TTR: Tracer:tracee ratio

## References

[1] Stinkens R, Goossens GH, Jocken JWE, Blaak EE (2015). Targeting fatty acid metabolism to improve glucose metabolism. Obes Rev.

[2] Goossens GH (2008). The role of adipose tissue dysfunction in the pathogenesis of obesity-related insulin resistance. Physiol Behav.

[3] Ebbert J, Jensen M (2013). Fat depots, free fatty acids, and dyslipidemia. Nutrients.

[4] Mittendorfer B (2011). Origins of metabolic complications in obesity: adipose tissue and free fatty acid trafficking. Curr Opin Clin Nutr Metab.

[5] Mensink M, Blaak EE, van Baak MA (2001). Plasma free fatty acid uptake and oxidation are already diminished in subjects at high risk for developing type 2 diabetes. Diabetes.

[6] Dubé JJ, Coen PM, DiStefano G (2014). Effects of acute lipid overload on skeletal muscle insulin resistance, metabolic flexibility, and mitochondrial performance. Am J Physiol Endocrinol Metab.

[7] Schrauwen P, Schrauwen-Hinderling V, Hoeks J, Hesselink MKC (2010). Mitochondrial dysfunction and lipotoxicity. Biochim Biophys Acta.

[8] Coen PM, Goodpaster BH (2012). Role of intramyocelluar lipids in human health. Trends Endocrinol Metab.

[9] Samuel VT, Shulman GI (2012). Mechanisms for insulin resistance: common threads and missing links. Cell.

[10] Blaak EE, Wagenmakers AJ, Glatz JF (2000). Plasma FFA utilization and fatty acid-binding protein content are diminished in type 2 diabetic muscle. Am J Physiol Endocrinol Metab.

[11] Bickerton AST, Roberts R, Fielding BA (2008). Adipose tissue fatty acid metabolism in insulin-resistant men. Diabetologia.

[12] Jocken JWE, Goossens GH, van Hees AMJ (2008). Effect of beta-adrenergic stimulation on whole-body and abdominal subcutaneous adipose tissue lipolysis in lean and obese men. Diabetologia.

[13] Hodson L, Bickerton AST, McQuaid SE (2007). The contribution of splanchnic fat to VLDL triglyceride is greater in insulin-resistant than insulin-sensitive men and women: studies in the postprandial state. Diabetes.

[14] Riemens SC, Sluiter WJ, Dullaart RP (2000). Enhanced escape of non-esterified fatty acids from tissue uptake: its role in impaired insulin-induced lowering of total rate of appearance in obesity and Type II diabetes mellitus. Diabetologia.

[15] Nielsen S, Karpe F (2012). Determinants of VLDL-triglycerides production. Curr Opin Lipidol.

[16] Choi SH, Ginsberg HN (2011). Increased very low density lipoprotein (VLDL) secretion, hepatic steatosis, and insulin resistance. Trends Endocrinol Metab.

[17] Bickerton AST, Roberts R, Fielding BA (2007). Preferential uptake of dietary fatty acids in adipose tissue and muscle in the postprandial period. Diabetes.

[18] Ruge T, Hodson L, Cheeseman J (2009). Fasted to fed trafficking of fatty acids in human adipose tissue reveals a novel regulatory step for enhanced fat storage. J Clin Endocrinol Metab.

[19] van Hees AMJ, Jans A, Hul GB (2011). Skeletal muscle fatty acid handling in insulin resistant men. Obesity.

[20] Goossens G, Moors C, Jocken J (2016). Altered skeletal muscle fatty acid handling in subjects with impaired glucose tolerance as compared to impaired fasting glucose. Nutrients.

[21] Jans A, Konings E, Goossens GH (2012). PUFAs acutely affect triacylglycerol-derived skeletal muscle fatty acid uptake and increase postprandial insulin sensitivity. Am J Clin Nutr.

[22] Bergström J, Hermansen L, Hultman E, Saltin B (1967). Diet, muscle glycogen and physical performance. Acta Physiol Scand.

[23] Bosma M, Kersten S, Hesselink MKC, Schrauwen P (2012). Re-evaluating lipotoxic triggers in skeletal muscle: relating intramyocellular lipid metabolism to insulin sensitivity. Prog Lipid Res.

[24] Moors CCM, van der Zijl NJ, Diamant M (2012). Impaired insulin sensitivity is accompanied by disturbances in skeletal muscle fatty acid handling in subjects with impaired glucose metabolism. Int J Obes (Lond).

[25] Samuel VT, Petersen KF, Shulman GI (2010). Lipid-induced insulin resistance: unravelling the mechanism. Lancet.

[26] Hiukka A, Fruchart-Najib J, Leinonen E (2005). Alterations of lipids and apolipoprotein CIII in very low density lipoprotein subspecies in type 2 diabetes. Diabetologia.

[27] Kersten S (2014). Physiological regulation of lipoprotein lipase. Biochim Biophys Acta.

[28] Heath RB, Karpe F, Milne RW (2003). Selective partitioning of dietary fatty acids into the VLDL TG pool in the early postprandial period. J Lipid Res.

[29] Heath RB, Karpe F, Milne RW (2007). Dietary fatty acids make a rapid and substantial contribution to VLDL-triacylglycerol in the fed state. Am J Physiol Endocrinol Metab.

[30] Yost TJ, Jensen DR, Haugen BR, Eckel RH (1998). Effect of dietary macronutrient composition on tissue-specific lipoprotein lipase activity and insulin action in normal-weight subjects. Am J Clin Nutr.

[31] Goldberg IJ, Eckel RH, Abumrad NA (2009). Regulation of fatty acid uptake into tissues: lipoprotein lipase- and CD36-mediated pathways. J Lipid Res.

[32] Sparks LM, Bosma M, Brouwers B (2014). Reduced incorporation of fatty acids into triacylglycerol in myotubes from obese individuals with type 2 diabetes. Diabetes.

[33] Montell E, Turini M, Marotta M (2001). DAG accumulation from saturated fatty acids desensitizes insulin stimulation of glucose uptake in muscle cells. Am J Physiol Endocrinol Metab.

[34] Perreault L, Bergman BC, Hunerdosse DM (2009). Inflexibility in intramuscular triglyceride fractional synthesis distinguishes prediabetes from obesity in humans. Obesity.

[35] Forouhi NG, Koulman A, Sharp SJ, Imamura F (2014). Differences in the prospective association between individual plasma phospholipid saturated fatty acids and incident type 2 diabetes: the EPIC-InterAct case. Lancet Diabetes Endocrinol.

[36] Jenkins B, West J, Koulman A (2015). A review of odd-chain fatty acid metabolism and the role of pentadecanoic acid (C15:0) and heptadecanoic acid (C17:0) in health and disease. Molecules.

[37] Andersson A, Nälsén C, Tengblad S, Vessby B (2002). Fatty acid composition of skeletal muscle reflects dietary fat composition in humans. Am J Clin Nutr.

[38] Chaurasia B, Summers SA (2015). Ceramides – lipotoxic inducers of metabolic disorders. Trends Endocrinol Metab.

[39] Borkman M, Storlien LH, Pan DA (1993). The relation between insulin sensitivity and the fatty-acid composition of skeletal-muscle phospholipids. N Engl J Med.

[40] Baur LA, OʼConnor J, Pan DA (1998). The fatty acid composition of skeletal muscle membrane phospholipid: its relationship with the type of feeding and plasma glucose levels in young children. Metabolism.

[41] Bonora E, Targher G, Alberiche M (2000). Homeostasis model assessment closely mirrors the glucose clamp technique in the assessment of insulin sensitivity: studies in subjects with various degrees of glucose tolerance and insulin sensitivity. Diabetes Care.

[42] DeFronzo RA, Jacot E, Jequier E (1981). The effect of insulin on the disposal of intravenous glucose. Results from indirect calorimetry and hepatic and femoral venous catheterization. Diabetes.

